# Gene Expression Analyses in Models of Rosiglitazone-Induced Physiological and Pathological Mineralization Identify Novel Targets to Improve Bone and Vascular Health

**DOI:** 10.3390/cells12202462

**Published:** 2023-10-16

**Authors:** Claudia Bruedigam, Johannes P. T. M. van Leeuwen, Jeroen van de Peppel

**Affiliations:** Department of Internal Medicine, Erasmus MC, Wytemaweg 80, 3015 CN Rotterdam, The Netherlands; claudia.bruedigam@qimrberghofer.edu.au (C.B.); h.vandepeppel@erasmusmc.nl (J.v.d.P.)

**Keywords:** osteoblasts, mesenchymal stem cells, vascular smooth muscle, rosiglitazone, PPAR-gamma, vascular calcification, gene expression profiling, cell differentiation

## Abstract

Clinical studies revealed detrimental skeletal and vascular effects of the insulin sensitizer rosiglitazone. We have shown earlier that rosiglitazone accelerates osteoblast differentiation from human mesenchymal stem cells (hMSC) at the expense of increased oxidative stress and cell death. In calcifying human vascular cells, rosiglitazone stimulates pathological mineralization, an effect diminished by the antioxidant resveratrol. Here, we aimed to elucidate transcriptional networks underlying the rosiglitazone-enhanced mineralization phenotype. We performed genome-wide transcriptional profiling of osteogenic hMSCs treated with rosiglitazone for short-term periods of 1 up to 48 h during the first two days of differentiation, a phase that we show is sufficient for rosiglitazone stimulation of mineralization. Microarray-based mRNA expression analysis revealed 190 probes that were differently expressed in at least one condition compared to vehicle-treated control. This rosiglitazone gene signature contained well-known primary PPAR targets and was also endogenously regulated during osteogenic hMSC differentiation and osteoblast-like differentiation of vascular smooth muscle cells (VSMCs) into calcifying vascular cells (CVCs). Comparative analysis revealed rosiglitazone targets that were commonly enriched in osteoblasts and CVCs or specifically enriched in either osteoblasts or CVCs. Finally, we compared expression patterns of CVC-specific genes with patient expression data from carotid plaque versus intact adjacent tissue, and identified five rosiglitazone targets to be differentially regulated in CVCs and carotid plaque but not osteoblasts when compared to their non-mineralizing counterparts. These targets, i.e., PDK4, SDC4, SPRY4, TCF4 and DACT1, may specifically control extracellular matrix mineralization in vascular cells, and hence provide target candidates for further investigations to improve vascular health.

## 1. Introduction

Rosiglitazone is a synthetic peroxisome proliferator-activated receptor gamma (PPAR-gamma) agonist belonging to the thiazolidinedione class of compounds with insulin-sensitizing and blood glucose- and lipid-lowering activities. Despite these beneficial metabolic actions, serious cardiovascular side effects and also detrimental skeletal effects have been reported for rosiglitazone in epidemiological studies [1,2,3,4,5,6]. Both physiological and pathological mineralization (reflecting atherosclerosis calcification) can be modeled in vitro. When supplemented with dexamethasone and β-glycerophosphate, human mesenchymal stem cells (MSCs) and also vascular smooth muscle cells (VSMCs) differentiate into mineralizing osteoblasts or calcifying vascular cells (CVCs), respectively. Physiological phases in osteoblast and osteoblast-like differentiation have been described: proliferation and basic matrix formation are initiated during phase 1; formation of calcifying matrix occurs during phase 2; apoptosis and mineralization start during phase 3 [7]. Several key transcription factors have been described that are necessary for the occurrence of osteoblast and osteoblast-like differentiation phases [8,9,10,11,12,13,14,15].

Rosiglitazone accelerates osteoblast differentiation from human mesenchymal stem cells (hMSCs) that is ultimately followed by excessive oxidative stress and osteoblast death, but eventually increases extracellular matrix mineralization [16]. In osteoblast-like differentiating VSMC cultures, rosiglitazone enhances extracellular matrix mineralization, which can be prevented by the antioxidant resveratrol [17]. In addition, others have shown that rosiglitazone inhibits osteoblast differentiation of mouse bone marrow cells and increases adipogenesis [18]. Although research so far has revealed novel insights into the relationships between rosiglitazone and cardiovascular [2,3] events as well as the clinically observed reduced bone quality, underlying molecular mechanisms have remained elusive.

In this study, we aimed to (1) investigate whether a specific differentiation phase is critical for initiating the rosiglitazone—accelerated osteoblast differentiation phenotype, (2) identify the transcriptional networks underlying the observed rosiglitazone-accelerated osteoblast differentiation phenotype and (3) compare the revealed rosiglitazone-mediated changes in the transcriptome with endogenous osteogenic and osteoblast-like patterns of gene expression. Comprehensive bioinformatics analyses were used to link the obtained results to osteoblast-like differentiation of VSMC and cardiovascular events.

## 2. Materials and Methods

### 2.1. Cell Culture

Human bone marrow-derived mesenchymal stem cells (hMSC; Lonza, Basel, Switzerland) were cultured as described previously [7]. MSCs were derived from two different donors and passages six and seven were used for the experiments. Adipogenic differentiation was induced by culturing hMSCs (5000 cell/cm^2^) in differentiation medium that was supplemented with 100 nM dexamethasone, 500 μM 3-isobutyl-1-methylxanthine (IBMX) and 60 μM indomethacin.

### 2.2. Mineralization and DNA Assays

Calcium and DNA measurements were performed as described previously [7].

### 2.3. Quantification of mRNA Expression

RNA isolation, cDNA synthesis and quantitative RT-PCR (Q-RT-PCR) were carried out as described previously [19]. Primer sequences as well as their concentrations are listed in Supplementary Table S1.

### 2.4. Illumina Gene Chip-Based Gene Expression

Illumina HumanHT-12 v3 BeadChip (Illumina, Inc., San Diego, CA, USA) human whole-genome expression arrays were used. RNA integrity of isolated RNA was assessed by RNA 6000 Nano assay on a 2100 Bioanalyzer (Agilent Technologies, Santa Clara, CA, USA). The RNA of two biological replicates for each condition was analyzed. The Illumina TotalPrep RNA Amplification Kit (Ambion, Austin, TX, USA) was used for RNA amplification of each sample according to manufacturer’s instructions. In short, T7 oligo(dT) primer was used to generate single-stranded cDNA followed by a second strand synthesis to generate double-stranded cDNA. In vitro transcription was performed to synthesize biotin-labeled cRNA using T7 RNA polymerase. The cRNA was column purified and checked for quality by RNA 6000 Nano assay. A total of 750 ng of cRNA was hybridized for each array using standard Illumina protocol with streptavidin-Cy3 (GE healthcare, Chicago, IL, USA) being used for detection. Slides were scanned on an iScan and analyzed using GenomeStudio (both from Illumina, Inc., San Diego, CA, USA). Raw data were background-subtracted using GenomeStudioV2010.1 (Gene expression module 1.6.0) and processed using the Bioconductor R2.10.0 lumi-package [20]. Data were variance stabilization-transformed and quantile-normalized. Differentially expressed probes were identified using Bioconductor package ‘limma’ [21].

### 2.5. Data Analyses

Publicly available gene expression datasets were downloaded from Gene Expression Omnibus (https://www.ncbi.nlm.nih.gov/geo/ (accessed on 1 October 2022)) under the accession number GSE37558 for calcifying vascular smooth muscle cells and osteoblasts, and GDS5083 for carotid artery atheroma datasets. The rosiglitazone gene expression dataset has been deposited into GEO under accession number GSE67518.

For gene ontology (GO) analysis, selected official gene symbols were analyzed using the 2022 Database for Annotation, Visualization and Integrated Discovery (DAVID) [22] hosted by the National Institute of Allergy and Infectious Diseases (NIAID), NIH (https://david.ncifcrf.gov/tools.jsp (accessed on 1 October 2022)) using *p* < 0.05 and a cut-off of at least 5 counts (=genes) per GO term.

Unsupervised hierarchical clustering of the log2 fold changes of expression intensities of probes was carried out using Genepattern (https://www.genepattern.org (accessed on 1 October 2022)). Dendrograms are displayed next to the respective rows and columns, and data shown by the colors were row-normalized.

Principal component analysis was carried out using Genepattern as well. As input, log2 expression changes of the rosiglitazone signature (190 probes) were used compared to MSC d0 condition. Genepattern was also used to perform gene set enrichment analysis (GSEA). Normalized gene expression intensities without filtering were used, and each probe was collapsed into a single vector per gene that was then identified by its official gene symbol. For each GSEA, 1000 permutations were performed. Based on PCA analyses [23] for GSEA of osteoblast differentiation, d0 and d2 were grouped (group1, “undifferentiated”) and compared with osteoblasts from all later timepoints (group 2, “differentiated”). For GSEA of pathological mineralization, VSMCs at d0 (group 1, “undifferentiated”) were compared with CVCs from all timepoints available (group 2, “differentiated”). An FDR *q*-value of <0.1 was used to define statistically significant enrichment.

Ingenuity Pathway Analysis (IPA) was used to perform upstream regulator analysis [24] and to generate gene regulatory networks around potential key genes. For upstream regulator analysis activation, z-scores of +2 and −2 were used as cut-offs for predicted activators or inhibitors, respectively. For upstream regulator analyses of the four identified expression pattern clusters, we focused on those in IPA classified as transcription regulators or liganded nuclear receptors. Interaction between the identified transcriptional regulators of the expression pattern clusters was established using STRING analyses (https://string-db.org (accessed on 1 May 2023)) [25]. The interaction source used was experiments and the networks indicate both functional and physical interaction. Significant PPI enrichment score means that the genes/proteins have more interactions among themselves than what would be expected for a random set of genes/proteins of the same size and degree distribution drawn from the genome. Such an enrichment indicates that the proteins are at least partially biologically connected, as a group. For IPA-generated gene regulatory networks of combined clusters 1 and 2 (up-regulated genes) as well as for clusters 3 and 4 (down-regulated genes), the two top networks were merged and direct up- and downstream connections were generated from an expression dataset filtered based on differential expression with *p* < 0.05. Quantitative expression changes were overlayed with upregulation shown in red and downregulation shown in green. Clusters 1 and 2 and clusters 3 and 4 were analyzed by IPA for association with diseases and functions.

Connectivity map was used as independent compound prediction analysis (CMAP; https://www.broadinstitute.org/cmap/ (accessed on 1 September 2022)). This approach uses data from about 6000 microarray experiments to correlate gene expression levels with about 1300 different drugs tested in four human cell lines (i.e., HL60, MCF7, SKMEL5 and PC3). The rosiglitazone clusters 1 and 2 were used as UP signature, and clusters 3 and 4 as DOWN signature. The obtained results were filtered based on *p* < 0.05.

Hypergeometric probability test was carried out using GeneProf software (http://www.geneprof.org/GeneProf/tools/hypergeometric.jsp (accessed on 1 September 2018)).

All other statistical analyses were carried out using Prism v6 (GraphPad Software, Boston, MA, USA). Comparisons with *p* < 0.05 determined by Student’s *t* test were defined as statistically significant.

## 3. Results

### 3.1. Rosiglitazone-Mediated Acceleration of Mineralization Is Initiated during the Early Osteoblast Differentiation Phase

**Figure 1:**
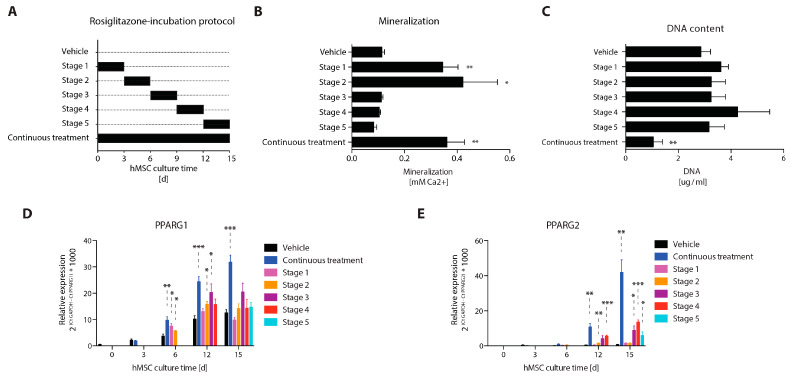
The stimulatory effect of rosiglitazone on mineralization is dependent on the early osteoblast differentiation phase. (**A**) Graphical representation of the rosiglitazone incubation protocol. Human bone marrow-derived MSCs were cultured on osteogenic differentiation medium supplemented with 10 uM rosiglitazone during the timepoints as displayed on the *x*-axis. (**B**) Quantification of mineralization and (**C**) DNA content during the various differentiation stages, continuous or vehicle-treated control conditions as depicted on the *y*-axis. Quantitative-RT-PCR analysis of (**D**) PPARG1 and (**E**) PPARG2. * *p* < 0.05; ** *p* < 0.01; *** *p* < 0.001; 6 replicates from two independent experiments. Statistics according to Student’s *t* test of the respective rosiglitazone condition compared to vehicle from the same timepoint.

We investigated whether a particular osteogenic differentiation phase is important for mediating the stimulatory effect of rosiglitazone on mineralization. Human MSC cultures were supplemented with 10 µM rosiglitazone or respective control for an interval of each three days during different phases of osteogenic differentiation (Figure 1A). ECM mineralization was quantified at day 15 of culture. Interestingly, rosiglitazone treatment during the first three days was already sufficient to stimulate ECM mineralization to a level similar to that observed by continuous treatment (Figure 1B). Rosiglitazone treatment from day 3 until day 6 revealed a similar effect, whereas treatment during any time interval from day 6 until day 15 did not lead to any changes in ECM mineralization (Figure 1B). The increased mineralization levels were not a result of increased cell numbers (Figure 1C). PPARG1 and PPARG2 expression levels were significantly increased upon continuous rosiglitazone treatment, whereas short-term rosiglitazone incubations showed only transient stimulatory effects on PPARG expression (Figure 1D,E).

### 3.2. Rosiglitazone Regulates Well-Characterized PPAR-Gamma Target Genes during the Early Osteoblast Differentiation Phase

**Figure 2:**
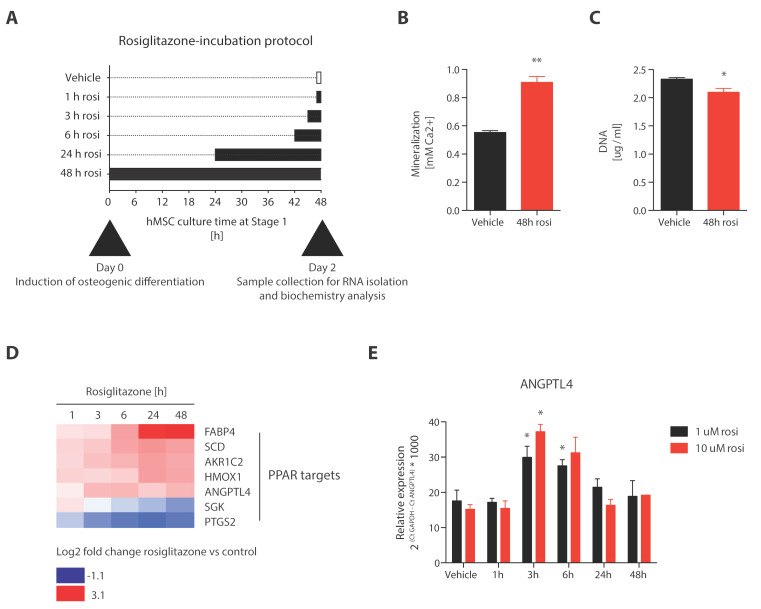

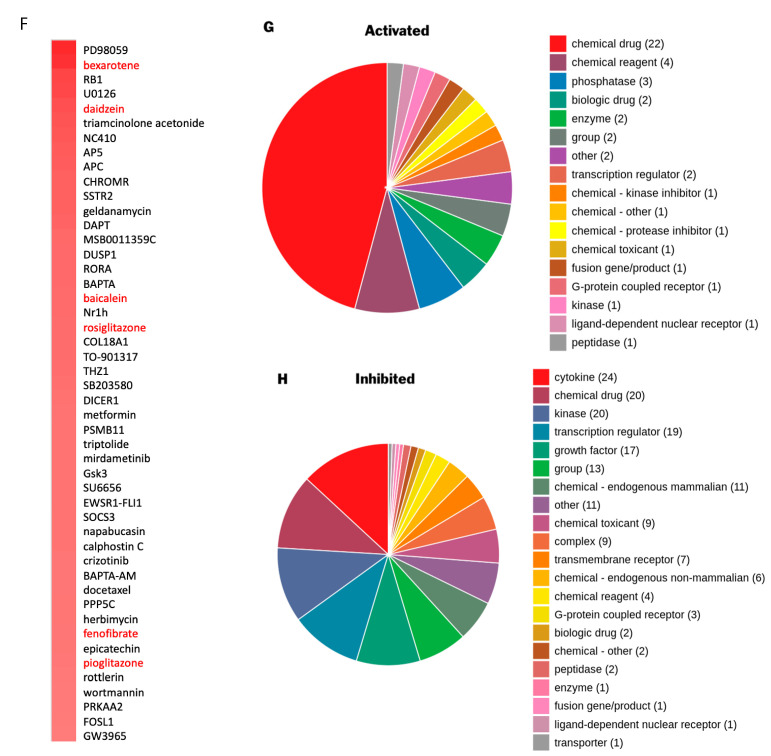
Rosiglitazone regulates well-characterized PPAR-gamma target genes during the early osteoblast differentiation phase. (**A**) Graphical display of the rosiglitazone incubation protocol. Osteogenic human bone-marrow derived MSCs were cultured in osteogenic medium and supplemented with 10 uM rosiglitazone during the time as depicted on the *x*-axis. The control condition was treated with vehicle for 1 h before harvest. (**B**) Mineralization and (**C**) DNA content of the cultures incubated with rosiglitazone for 48 h (d0–2) compared to vehicle control analyzed on day 15. (**D**) Heatmap displaying the microarray-based expression fold changes of well-characterized PPAR target genes in the rosiglitazone treated hMSC conditions compared to controls as described in (**A**). (**E**) Quantification of the expression of the confirmed PPAR-gamma target gene ANGPTL4 by q-RT-PCR analysis in samples from 2 independent experiments generated similarly to as described in (**A**). * *p* < 0.05. ** *p* < 0.01 *n* = 6. Expression levels were calculated relative to GAPDH. (**F**) Upstream regulator analysis of the rosiglitazone-regulated gene signature (q < 0.05) using Ingenuity Pathway Analysis software (IPA). Heatmap displays activation z-scores. Compound names are displayed on the right of the corresponding heat block. Only compounds with a positive enrichment score (>2) are shown. Classification and distribution of the identified 48 activated (**G**) and 183 inhibited (**H**) upstream regulators into chemicals (dark blue) and biomolecules (red).

To identify rosiglitazone-targeted transcriptional networks, we performed genome-wide transcriptional profiling of 10 µM rosiglitazone- and control-supplemented hMSC cultures during the initially observed important early (0–3 days) phase. The detailed experimental layout is depicted in Figure 2A. Human MSC cultures were supplemented with rosiglitazone for 1, 3, 6, 24, or 48 h before harvest at 48 h (Figure 2A). This narrow time window was already sufficient for rosiglitazone to significantly enhance mineralization (Figure 2B). This stimulation of mineralization was not the result of increased cell numbers (Figure 2C). We found that the expression intensities of 190 probes, representing 185 transcripts, were significantly different from vehicle-treated control (q < 0.05, Supplementary Table S2) during at least one timepoint. Five genes were represented by two different probes each (PTGS2, IL8, GADD45A, ASS1, ALDH1A3). Thus, in total, expression levels of 185 transcripts were changed significantly upon rosiglitazone treatment. Confirmed primary PPAR target genes were included in this gene signature, and detailed analyses of expression fold changes in the different rosiglitazone versus control conditions revealed distinct PPAR target gene expression patterns (Figure 2D). The expression levels of fatty acid binding protein 4 (FABP4), stearoyl-CoA desaturase (SCD), aldo-keto reductase family 1 member C2 (AKR1C2), heme oxygenase 1 (HMOX1) and angiopoietin 4 (ANGPTL4) were increased with extended rosiglitazone incubation time compared to vehicle controls, whereas the expression levels of serum/glucocorticoid regulated kinase 1 (SGK1) and prostaglandin-endoperoxide synthase 2 (PTGS2) were reduced upon prolonged incubation time with rosiglitazone (Figure 2D). The observed expression pattern of ANGPTL4 was confirmed by quantitative RT-PCR (Figure 2E). To further investigate whether the revealed rosiglitazone gene signature has been associated with stimulated PPAR signaling in a broader range of biological contexts, we applied two different bioinformatics approaches. Firstly, we performed upstream regulator analysis using Ingenuity Pathway Analysis software (https://digitalinsights.qiagen.com/products-overview/discovery-insights-portfolio/analysis-and-visualization/qiagen-ipa/?cmpid=QDI_GA_IPA&gad_source=1&gclid=Cj0KCQjwj5mpBhDJARIsAOVjBdqO3NDZfbzQpgb07goycFuDJNcZfw1qtTa1e6sp62WfyLD4JP7ZScAaAm2CEALw_wcB, accessed on 3 September 2023, Qiagen, Hilden, Germany, release June 2023). We found that the thiazolidinediones rosiglitazone and pioglitazone, and the PPAR agonists fenofibrate and daidzein, as well as bexarotene and baicalein, were amongst the upstream predicted regulatory molecules with the strongest positive activation z-scores (Figure 2F, Supplementary Table S3). Most upstream regulators with activation z-scores of 2 or higher included chemicals, whereas biomolecules represented only a minor part. (Figure 2G, Supplementary Table S3). In contrast, upstream regulators with negative activation z-scores of less than −2 predicting inhibition mainly comprised biomolecules including transcription regulators, cytokines, kinases and growth factors (Figure 2H, Supplementary Table S3). Finally, we compared the revealed rosiglitazone gene signature from the current study with publicly available datasets using “Connectivity Map”. The two identified compounds with a positive score > 99 were two compounds inhibiting tyrosine kinase signaling (Supplementary Table S4). Eight compounds had a negative score < −99. Interestingly, five of these compounds are ATPase inhibitors (Supplementary Table S4).

In summary so far, genome-wide expression profiling of rosiglitazone-treated osteogenic human MSC cultures has revealed a distinct gene signature that contains well-characterized PPAR target genes and is associated with PPAR stimulation in a broad range of biological systems. Furthermore, compounds with high similarity to the effects of rosiglitazone on gene expression were identified.

### 3.3. Rosiglitazone Target Genes Cluster into Separate Expression Pattern Groups That Underlie Discrete Upstream Regulatory Processes

**Figure 3:**
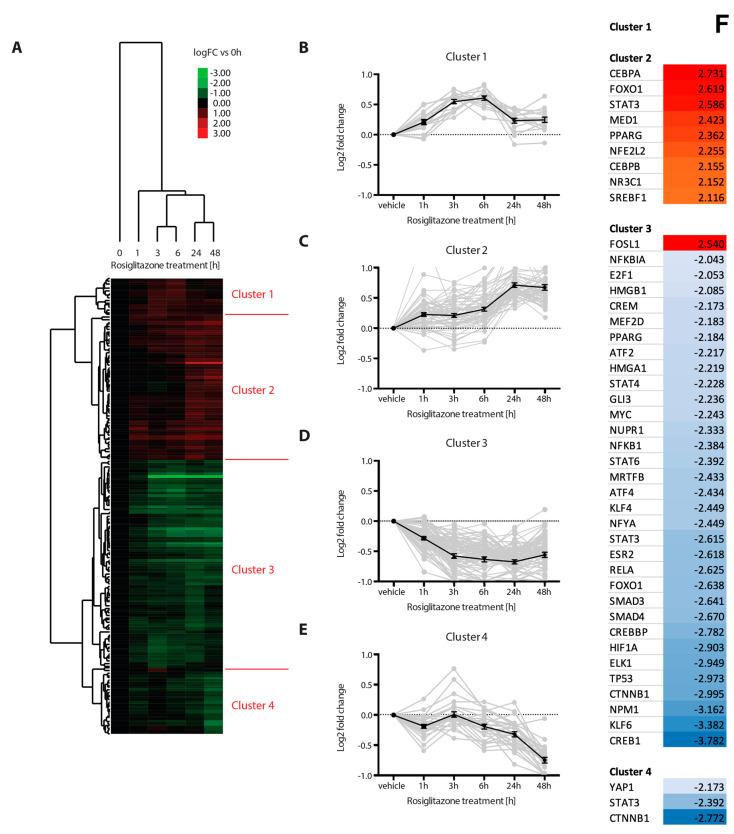

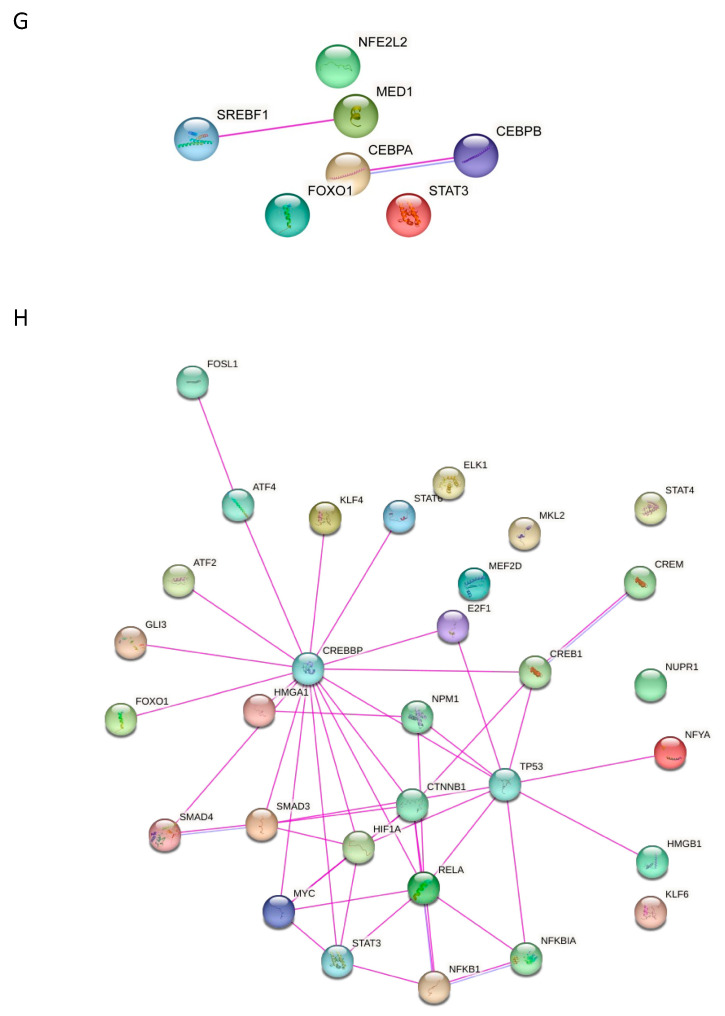
Rosiglitazone target genes cluster into separate expression pattern groups that underlie discrete upstream regulatory processes. (**A**) Heatmap displaying the results obtained from hierarchical clustering analysis. Probesets included were selected based on q < 0.05 in at least one condition compared to control. Blue: low expression fold change; Red: high expression fold change compared to controls; row-normalized. The data were generated from 3 wells in duplicate for each condition. (**B**–**E**) Average expression patterns of the four clusters. Data represented are mean ± SE log fold change of each rosiglitazone condition compared to controls. (**F**) Upstream regulator analysis of the four individual clusters from Figure 3A using Ingenuity Pathway Analysis. Transcription regulators were selected; activation and inhibition predication z-score cut-off was +2.00 or −2.00, respectively. (**G**) Interaction network representation of cluster 2 transcriptional regulators. PPI enrichment score *p* = 0.0072. (**H**) Interaction network representation of cluster 3 transcriptional regulators. PPI enrichment score *p* < 1.0 × 10^−16^.

In order to gain further insights into the nature and dynamics of rosiglitazone-induced changes of the transcriptome in osteogenic human MSCs, we performed hierarchical clustering analysis of the identified rosiglitazone targets based on their fold changes of gene expression in each rosiglitazone incubation condition compared to vehicle control. We observed great similarity between 3 and 6 or 24 and 48 h time-points, respectively (Figure 3A). Unsupervised hierarchical clustering revealed a separation of gene expression patterns into four major groups (Figure 3A). Gene expression patterns of transcripts in cluster 1 were overall increased compared to vehicle controls, reaching a plateau during 3 and 6 h of incubation with rosiglitazone (Figure 3B). Transcript expression patterns in cluster 2 revealed an upregulation that was particularly pronounced during 24 and 48 h of incubation with rosiglitazone (Figure 3C). In total, clusters 1 and 2 were composed of 65 genes. Expression levels of rosiglitazone target genes were reduced compared to control conditions in both clusters 3 and 4 (Figure 3D,E). Expression levels were reduced during 3, 6, 24 and 48 h of rosiglitazone treatment compared to controls in cluster 3 (Figure 3D), whereas transcripts belonging to cluster 4 showed remarkable downregulation after 48 h of rosiglitazone incubation (Figure 3E). To investigate whether clustering of targets according to their expression patterns would reveal cluster-specific transcription factors orchestrating rosiglitazone-initiated responses, we performed upstream regulator prediction analysis using Ingenuity Pathway Analysis (Figure 3F). No transcriptional regulator for cluster 1 was identified, while in addition to PPARG, the activation of FOXO1, CEBPA, CEBPB, MED1, STAT3, NFE2L2, NR3C1 and SREBF1 transcription factors was predicted for cluster 2 (Figure 3F). Cluster 3 containing transcripts downregulated upon rosiglitazone treatment was predicted to result from activation of AP-1 signaling and inhibition of PPARG as well as of a large number of transcription factors, including SMAD, STAT, NfkB, E2F, WNT and cAMP response element binding protein signaling (Figure 3F). Finally, beta-catenin, STAT3 and YAP1 signaling are linked to cluster 4 (Figure 3F). STRING network analyses of cluster 2 and cluster 3 regulators show the functional and/or physical interaction of these transcriptional regulators (Figure 3G,H). For both clusters, the networks have a significant PPI enrichment score (cluster 2: *p* = 0.007; cluster 3: *p* < 1.0 × 10^−16^). This means that they have more interactions than expected and that the transcriptional regulators are at least partially biologically connected. In summary, we identified distinct clusters of rosiglitazone target genes based on their expression patterns that were reflecting distinct gene regulatory modules.

### 3.4. Rosiglitazone Target Clusters Are Annotated with Distinct Cellular Compartments, Molecular Functions and Biological Processes

**Figure 4:**
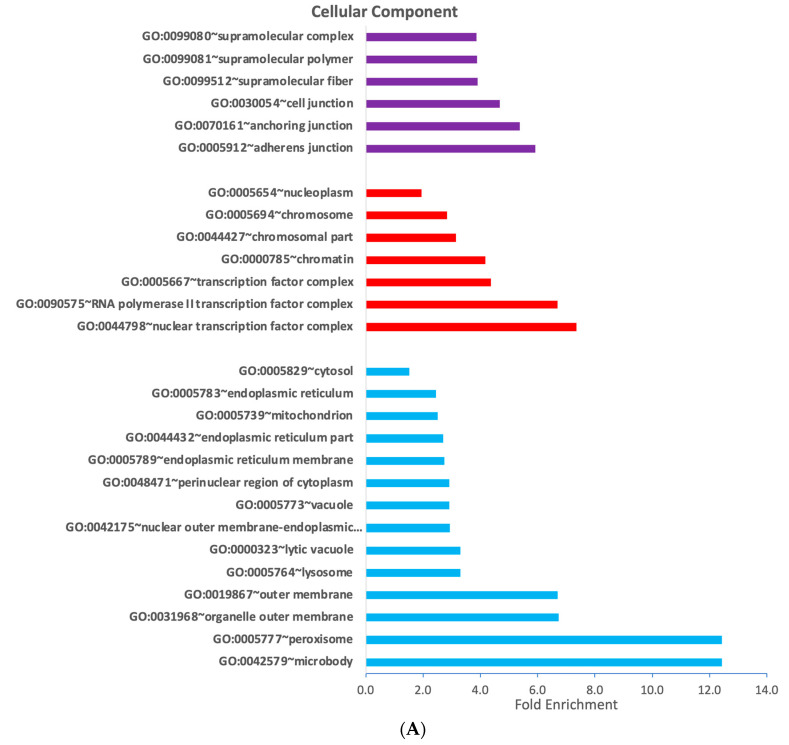

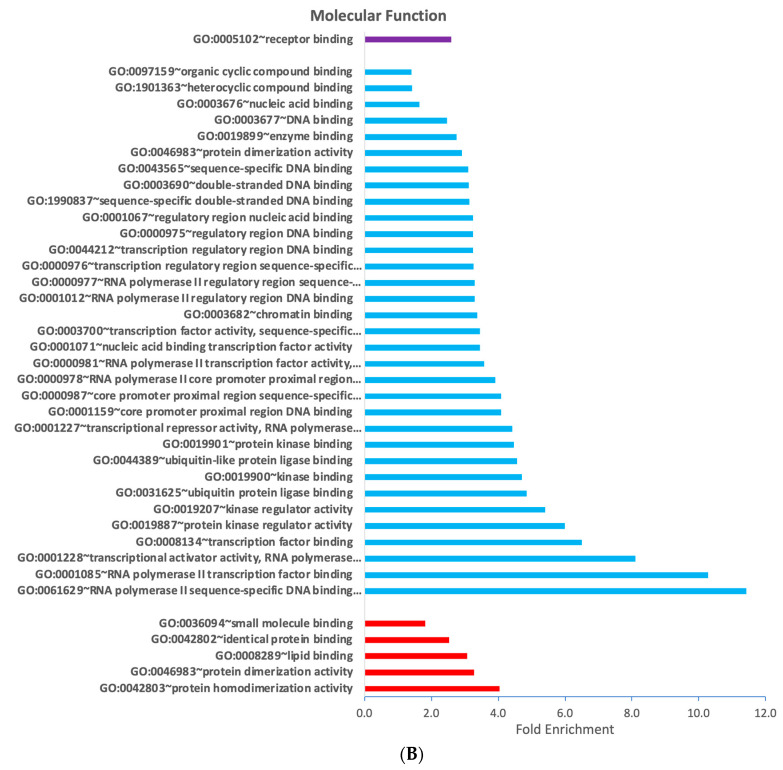
Rosiglitazone target clusters are annotated with distinct cellular compartments, molecular functions and biological processes. DAVID gene functional classification tool—based gene ontology analysis of the four individual clusters according to (**A**) cellular compartments and (**B**) molecular function. Annotations were selected based on *p* < 0.05.

In order to investigate whether the revealed gene regulatory modules would also reflect some functional distinction, we performed gene ontology analysis using the DAVID functional annotation tool. Regarding cellular compartments, we found that cluster 2 was enriched for cytosol, endoplasmic reticulum and cytosolic organelles (Figure 4A). Clusters 3 and 4 were enriched for nucleus-associated compartments and cell junctions, respectively (Figure 4A, Supplementary Table S5). Regarding biological processes, we did not observe processes in cluster 1 with at least five counts (i.e., five genes in the gene list that belong to the gene category). Biological processes involved in lipid metabolism, apoptosis and cellular response to external perturbation are prominent in cluster 2 (Supplementary Table S6). Cell metabolism, gene transcription and biosynthesis are prominent biological processes in cluster 3, and wound healing and cell motility was enriched in cluster 4 (Supplementary Table S6). Regarding molecular functions, interaction and binding of proteins and lipids were identified in cluster 2 (Figure 4B, Supplementary Table S7). In cluster 3, gene expression regulatory pathways were mainly enriched, including RNA polymerase II regulation and activity and double-stranded DNA binding processes (Figure 4B, Supplementary Table S7), and receptor binding was enriched in cluster 4 (Figure 4B, Supplementary Table S7). In summary, we demonstrated that the distinct rosiglitazone target modules were annotated with distinct subcellular localizations and functions demonstrating an overall suppression of gene expression involved in direct transcriptional regulation, and an overall stimulation of gene expression involved in metabolism.

Analyses of the upregulated genes (clusters 1 and 2) and the downregulated genes (clusters 3 and 4) for association with Diseases and Functions in IPA delivered an intriguing observation. Organismal development, in particular Size of Body, showed the strongest increased and decreased activation score for the upregulated and downregulated genes, respectively (Supplementary Table S8). This is interesting considering the relationship between the skeleton and body size/height.

### 3.5. The Rosiglitazone Gene Signature Can Discriminate Differentiating hMSC and VSMC Cultures Based on Their Differentiation Status

**Figure 5:**
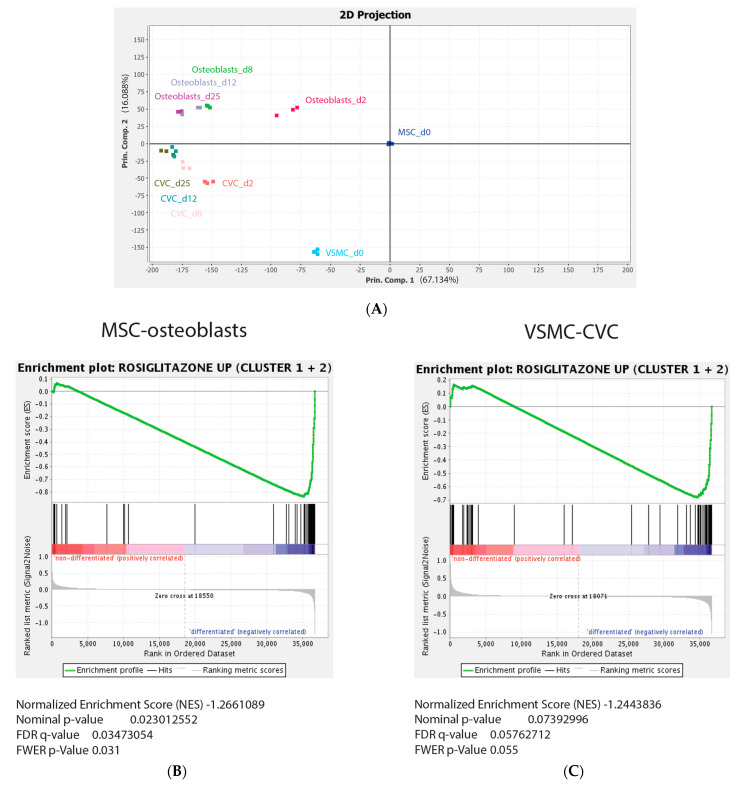
A rosiglitazone-targeted gene signature can discriminate differentiating hMSC and VSMC cultures based on their differentiation status. (**A**) Principal component analysis (PCA) of the rosiglitazone signature in osteoblasts differentiating from hMSCs and CVCs differentiating from VSMC cultures. (**B**,**C**) GSEA of clusters 1 and 2 from the rosiglitazone signature comparing differentiated osteoblasts with MSC and early (d2) osteoblast cultures (**B**), and comparing CVCs with VSMCs (**C**). (**B**,**C**) show GSEA results. The obtained negative enrichment scores indicate an enrichment of the cluster 1 and 2 signature in the “differentiated” groups in both osteogenic MSC and VSMC-CVC analyses. Statistical parameters for MSC—osteoblast GSEA were determined as the following: normalized enrichment score (−1.266), nominal *p*-value (0.02), FDR q-value (0.03), FWER *p*-value (0.03). For VSMC—CVC GSEA, the following statistical parameters were quantified: normalized enrichment score (−1.244), nominal *p*-value (0.07), FDR q-value (0.058), FWER *p*-value (0.055).

In order to further investigate whether the identified rosiglitazone gene signature would be important for osteogenic and osteoblast-like differentiation processes of hMSC or VSMC cultures, respectively, we performed principal component analysis of the rosiglitazone gene signature on previously published gene expression datasets from hMSC and VSMC cultures differentiating into osteoblasts or calcifying vascular cells (CVCs), respectively [23]. All 185 genes comprising the rosiglitazone signature were found to be present in these hMSC and VSMC datasets. We found that the undifferentiated MSC and VSMC cultures showed a clear difference, as indicated by the long distance between them on the two-dimensional PCA plot showing the first and second principal component, explaining 67% or 16% of the variance, respectively (Figure 5A). Interestingly, both differentiating osteoblasts and CVCs diverged from their non-mineralizing counterparts into the same direction, and the most differentiated osteoblast and CVC cultures showed greater similarity, as indicated by the reduced distance from each other (Figure 5A). The first principal component thus describes the differentiation, and the second principal component describes the cell type. We then added GSEA to learn about the directionality of gene expression changes of rosiglitazone targets during endogenous osteogenic differentiation. We found that the upregulated genes (clusters 1 and 2; Figure 3B,C) were significantly enriched in osteogenic differentiation (i.e., comparing the differentiated osteoblasts with undifferentiated gene expression profiles; Figure 5B), whereas no significant enrichment was found for the downregulated genes (clusters 3 and 4; Figure 3D,E). During osteogenic differentiation of VSMCs into CVCs, we found a marked borderline significant enrichment of rosiglitazone targets from clusters 1 and 2 (Figure 5C), and here also, no significant enrichment for clusters 3 and 4 was observed. In summary, these analyses have provided evidence for the presence of the upregulated rosiglitazone signature during osteogenic differentiation of MSC and VSMC, suggesting that rosiglitazone targets may be important components for the induction of both physiological and pathological mineralization.

### 3.6. Identification of Commonly and Specifically Enriched Rosiglitazone Targets during Physiological and Pathological Mineralization

**Figure 6:**
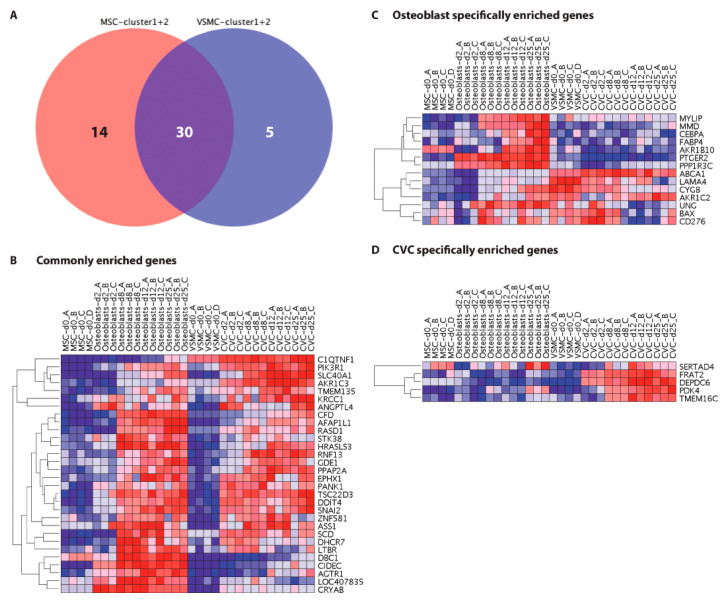
Identification of commonly and specifically enriched rosiglitazone targets during physiological and pathological mineralization. (**A**) VENN diagram of cluster 1 and 2 genes (Figure 2B,C) identified to be significantly enriched by GSEA analyses of osteoblasts versus MSCs (red) and of CVCs versus VSMCs (blue) (analyses shown in Figure 5). Heatmaps displaying expression patterns of the genes identified by GSEA and found to be (**B**) commonly enriched in osteoblasts and CVCs, (**C**) specifically enriched in osteoblasts, and (**D**) specifically enriched in CVCs. All data were row-normalized.

To investigate whether the rosiglitazone targets may be enriched specifically in either osteoblasts or CVCs, we generated a VENN diagram from the significantly enriched genes determined by GSEA as shown above (Figure 6A). This analysis revealed that the majority, i.e., 30 genes, were found to be commonly enriched in both physiological and pathological mineralization (Figure 6B), whereas only a small number of 14 and 5 genes were found to be specifically enriched in osteoblasts or CVCs, respectively (Figure 6C,D).

**Figure 7:**
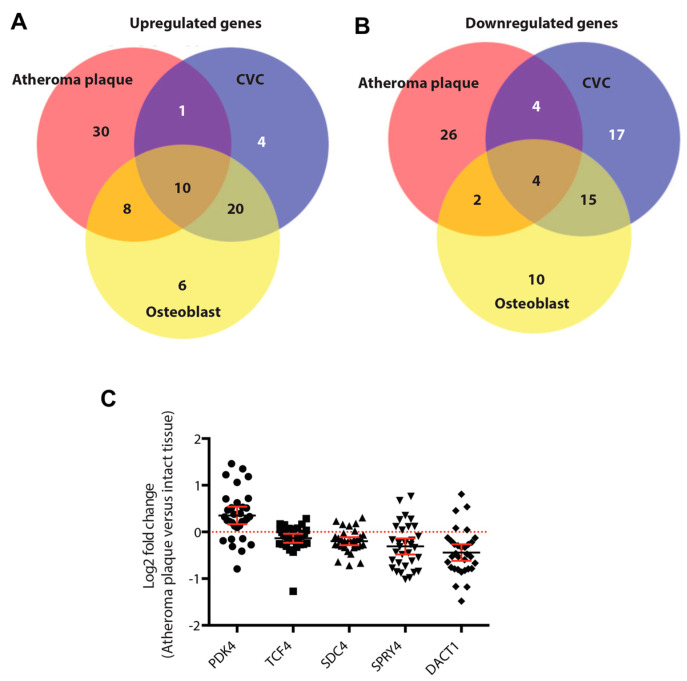

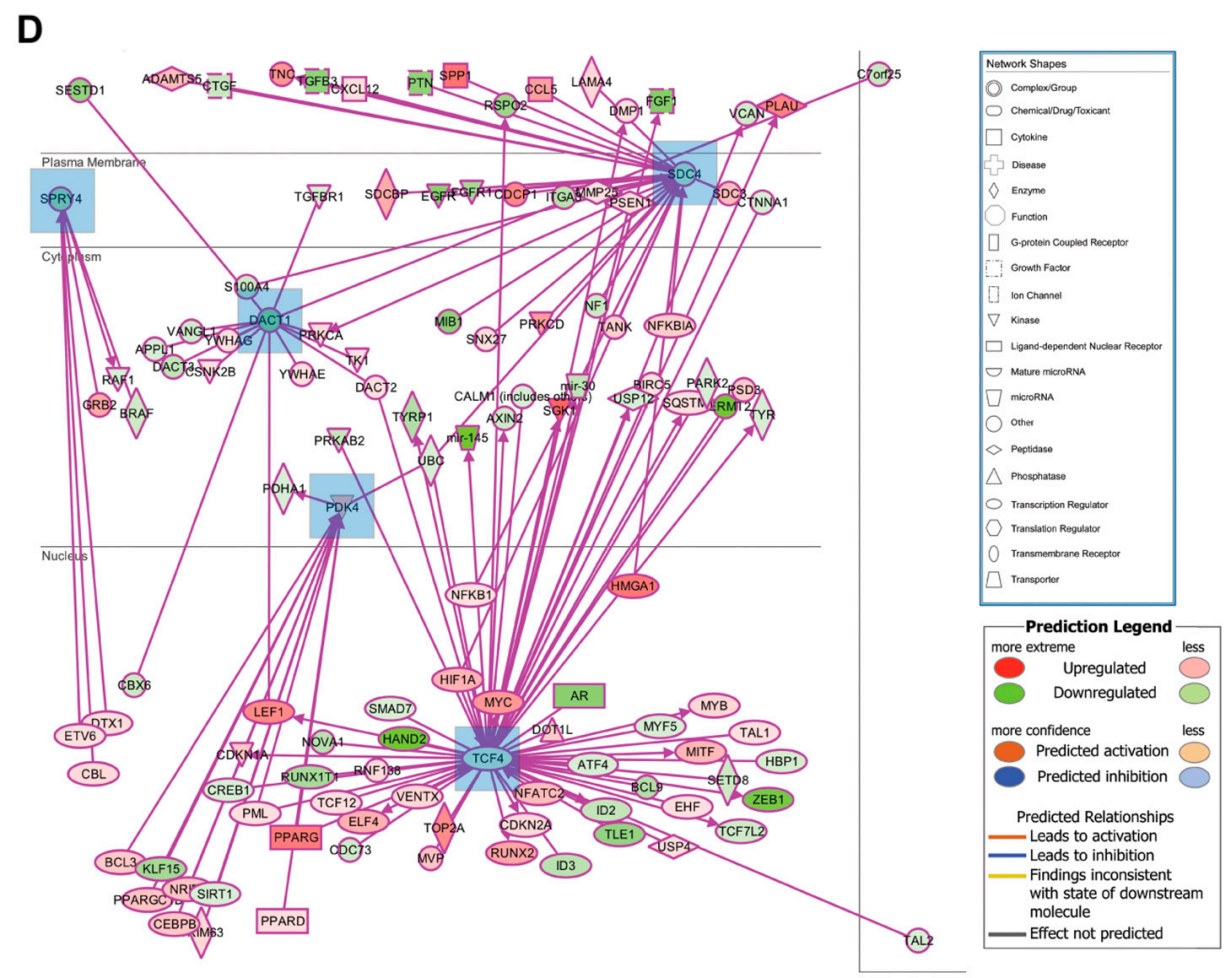
Identification of rosiglitazone targets in atherosclerosis. (**A**) Venn diagrams of the genes from the upregulated carotid plaque cluster and clusters 1 and 2 in osteoblasts and CVCs, and (**B**) of the genes from the downregulated carotid plaque cluster and clusters 3 and 4 in osteoblasts and CVCs. (**C**)Pairwise carotid plaque versus intact tissue expression changes of the identified 5 pathological mineralization–specific rosiglitazone targets PDK4, TCF4, SDC4, SPRY4 and DACT1. Data from each atherosclerotic patient are represented ±95% confidence interval. (**D**) Ingenuity Pathway Analysis-generated gene expression regulatory networks around the 5 key targets identified with overlaid expression changes from the patient dataset (carotid plaque versus intact tissue, cut-off *p* < 0.05).

We finally aimed to investigate whether the identified rosiglitazone gene signature would be detectable in patient-derived carotid plaque tissue [26]. From the 185 gene rosiglitazone signature, 148 were present in the carotid artery atheroma datasets. We found that 85 out of these 148 genes showed a significant (*p* < 0.05) difference in expression in carotid plaque tissue compared to intact adjacent tissue, a number that was well above chance according to hypergeometric probability test (*p* < 1.6 × 10^−12^). Next, comparative analysis (i.e., carotid plague versus intact tissue; differentiated osteoblasts versus undifferentiated MSCs, and CVCs versus VSMCs) of rosiglitazone genes was performed. Comparative analysis of the upregulated cluster revealed that 1 gene was also enriched specifically in CVCs, while 10 genes were also enriched in both osteoblasts and CVCs (Figure 7A). For the downregulated cluster, we identified four genes that were also specifically enriched in CVC, and four additional genes that were also enriched in both CVC and MSCs (Figure 7B). The gene found to be specifically upregulated in carotid plaque and CVC was identified as PDK4 encoding for pyruvate dehydrogenase kinase isoform 4, and the genes identified to be downregulated exclusively in carotid plaque and CVC but not physiological mineralization were identified as TCF4, SDC4, SPRY4 and DACT1 encoding for transcription factor 4, syndecan 4, sprout homolog 4 (Drosophila) and dishevelled binding antagonist of beta-catenin 1, respectively (Figure 7C). Finally, we investigated whether these five candidate genes identified for rosiglitazone-stimulated pathological mineralization were key components of gene regulatory networks. We therefore performed Ingenuity Pathway Analysis-based generation of gene regulatory networks starting from the five key genes and overlaid the revealed connected up- and downstream molecules with the gene expression data from carotid plaque versus adjacent intact tissue (Figure 7D). We found overall that the five key candidates identified were well connected with differentially regulated genes in carotid plaques, and hence may provide promising candidates to develop targeted therapies to improve vascular health.

## 4. Discussion

The current study has revealed three major findings. Firstly, short-term rosiglitazone treatment during the early phase of osteogenic hMSC differentiation is already sufficient to accelerate osteoblast differentiation and bone matrix mineralization that is observed two to three weeks later during culture. Secondly, a rosiglitazone gene signature detected during the observed important early phase can stratify osteoblast and osteogenic CVC cultures based on their differentiation status. And thirdly, the rosiglitazone gene signature is also present in carotid plaque. Five rosiglitazone targets (PDK4, TCF4, SDC4, SPRY4 and DACT1) are specifically dysregulated in pathological mineralization, providing a rationale for these targets to be validated in further studies to improve vascular health.

The data from the current study demonstrate that rosiglitazone initiates its acceleratory effects on differentiation during the initial phase of osteoblast differentiation. In line with this finding, a particularly high susceptibility of the early osteoblast differentiation phase [14] to environmental perturbations has been demonstrated recently in regard to oxygen tension [27], 1alpha, 25-dihydroxyvitamin D3 and interferon-beta [28]. Also, RUNX2 and WNT effects are time-dependent and particularly important during the early phase of osteoblast differentiation [15].

The finding that PPARG expression was lowest in early differentiation stages when rosiglitazone treatment initiated the strongest effects demonstrates that the level of receptor expression is not directly correlated with the effects of rosiglitazone on osteoblast differentiation and mineralization. This suggests that a low expression level of PPARG is already sufficient to mediate the rosiglitazone-initiated effects on differentiation and mineralization, or that at least parts of the effects may be mediated PPARG-independently. PPARG-independent effects of thiazolidinediones have been described earlier [29]; however, we have shown that knockdown of PPARG1 expression diminished, and overexpression of PPARG1 further increased osteoblast differentiation [16]. It is therefore conceivable that a low level of receptor is already sufficient. In support of this hypothesis, we demonstrated that the gene networks regulated by rosiglitazone contain well-characterized PPAR target genes and are also associated with PPAR stimulation in a broad range of other biological systems.

The compounds identified by upstream regulator prediction or connectivity map-based analysis of the rosiglitazone signature include, besides rosiglitazone itself, well-characterized PPAR agonists such as the additional thiazolidinediones pioglitazone, as well as natural PPAR ligands including fenofibrate and daidzein. Hence, these data confirm the sanity of the chosen approach to identify novel compounds with potential PPAR agonistic activity or similar working mechanisms, and may provide a starting point for drug repositioning [30]. The identified drugs represent various compound classes derived from natural or synthetic origins, and the variety of structures and functional annotations is in line with the role of PPARs as low-affinity nuclear receptors orchestrating a wide range of cellular processes including fatty acid catabolism, inflammatory responses, cell proliferation, apoptosis and differentiation by direct or indirect modes of gene expression regulation [31,32,33,34].

The fact that differentiated osteoblasts and CVCs show remarkable similarity based on their reduced distance by principal component analysis is striking as Alves and co-workers found that, despite some similarities, the transcriptomes of osteoblasts and CVCs were overall different, and the degree of difference did not change during the time course of differentiation [23]. The rosiglitazone gene signature can discriminate osteoblasts from MSCs and CVCs from VSMCs based on their differentiation status. Differentiated CVCs and osteoblasts are more like each other than undifferentiated MSCs and VSMCs. These results suggest that the rosiglitazone gene signature contains important regulators of extracellular matrix production and mineralization that are common between physiological and pathological conditions.

Despite the great overlap identified, we have focused our efforts on the identification of CVC-specific rosiglitazone targets considering the serious cardiovascular side effects that have been reported for rosiglitazone [3,4,6], as those may provide the most promising candidates to target pathological mineralization. We were able to identify five key genes differentially expressed in CVCs compared to VSMCs as well as in carotid plaque tissue compared to the respective adjacent intact tissue, showing the same directionality of gene expression change: PDK4, SDC4, SPRY4, TCF4 and DACT1. PDK4 is upregulated in CVCs and also overexpressed in carotid plaques. In line with this finding, the pyruvate dehydrogenase complex has been described as an emerging target for the treatment of metabolic syndrome. To maintain a steady-state level of adenosine triphosphate during the feed-fast cycle, cells need to utilize fatty acid and glucose efficiently that is controlled by the pyruvate dehydrogenase complex. PDK4 expression in cardiomyocytes decreases with age, which may be related to a change in cardiomyocyte energy metabolism at older age [35]. Lee et al. mentioned an important role for PDK4 in vascular mineralization including an upregulation of PDK4 and phosphorylation of the pyruvate dehydrogenase complex in cultured VSMCs and calcified vessels of patients with atherosclerosis [36]. In their mentioned work, PDK4 promoted osteogenic differentiation of VSMCs by phosphorylating SMAD1/5/8 and enhancing bone morphogenic protein 2 signaling [36].

The remaining four key genes identified (SDC4, TCF4, DACT1 and SPRY4) are downregulated in CVCs and also underexpressed in carotid plaques. SPRY4 is a member of the Spry protein family that can serve as feedback modulator of receptor tyrosine kinases, PI3K/Akt and MAPK/ERK signaling mechanisms. Interestingly, SPRY4 has been described recently as a suppressor of VSMC differentiation by antagonizing both MAPK/ERK and Akt signaling in vitro [37]. The downregulation of SPRY4 may facilitate the differentiation process of VSMCs that may, together with other stimuli, facilitate the differentiation into CVCs.

DACT1 is a member of the Dact protein family of multi-domain adaptor proteins that serve as a nodal point in regulating many cellular activities by regulating Wnt and Tgf-beta signaling [38]. However, potential roles of DACT1, SDC4 and TCF4 in pathological mineralization have not yet been described.

## 5. Conclusions

The current study provides evidence for a particularly high susceptibility to rosiglitazone-mediated perturbation of the osteoblast transcriptome during the early differentiation phase leading to accelerated osteoblast differentiation and mineralization. The identified rosiglitazone gene signature can discriminate non-mineralized VSMC, MSC and early osteoblast cultures from differentiated and mineralized osteoblast and CVC cultures and is associated with an increased mineralization phenotype. Comprehensive comparative analyses have revealed five rosiglitazone targets that are differentially regulated exclusively in pathological mineralization and hence serve as promising candidates to improve vascular health.

## Data Availability

The rosiglitazone gene expression data analyzed in this publication have been deposited in NCBI’s Gene Expression Omnibus and are accessible through GEO Series accession number GSE67518 (http://www.ncbi.nlm.nih.gov/geo/query/acc.cgi?acc=GSE67518 accessed on 5 October 2023).

## Supplementary Materials

The following supporting information can be downloaded at: https://www.mdpi.com/article/10.3390/cells12202462/s1, Table S1: Supplementary-table1.xlsx; Table S2: Supplementary-table2.xlsx; Table S3: Supplementary-table3.xlsx; Table S4: Supplementary-table4.xlsx; Table S5: Supplementary-table5.xlsx; Table S6: Supplementary-table6.xlsx; Table S7: Supplementary-table7.xlsx; Table S8: Supplementary-table8.xlsx.
